# Ethical framework for the detection, management and communication of incidental findings in imaging studies, building on an interview study of researchers’ practices and perspectives

**DOI:** 10.1186/s12910-017-0168-y

**Published:** 2017-02-06

**Authors:** Eline M. Bunnik, Lisa van Bodegom, Wim Pinxten, Inez D. de Beaufort, Meike W. Vernooij

**Affiliations:** 1 Department of Medical Ethics and Philosophy of Medicine, Erasmus MC, University Medical Centre Rotterdam, Wytemaweg 80, 3015 CN Rotterdam, The Netherlands; 2 Department of Morphology, Hasselt University, Agoralaan Gebouw D, BE 3590 Diepenbeek, Belgium; 3 Department of Epidemiology, Erasmus MC, University Medical Centre Rotterdam, Wytemaweg 80, 3015 CN Rotterdam, The Netherlands; 4 Department of Radiology and Nuclear Medicine, Erasmus MC, University Medical Centre Rotterdam, Wytemaweg 80, 3015 CN Rotterdam, The Netherlands

**Keywords:** Incidental findings, Research ethics, Imaging studies, Population imaging, Interview study, Ethical framework, Principle of reciprocity

## Abstract

**Background:**

As thousands of healthy research participants are being included in small and large imaging studies, it is essential that dilemmas raised by the detection of incidental findings are adequately handled. Current ethical guidance indicates that pathways for dealing with incidental findings should be in place, but does not specify what such pathways should look like. Building on an interview study of researchers’ practices and perspectives, we identified key considerations for the set-up of pathways for the detection, management and communication of incidental findings in imaging research.

**Methods:**

We conducted an interview study with a purposive sample of researchers (*n* = 20) at research facilities across the Netherlands. Based on a qualitative analysis of these interviews and on existing guidelines found in the literature, we developed a prototype ethical framework, which was critically assessed and fine-tuned during a two-day international expert meeting with bioethicists and representatives from large population-based imaging studies from the United Kingdom, Germany, Sweden and Belgium (*n* = 14).

**Results:**

Practices and policies for the handling of incidental findings vary strongly across the Netherlands, ranging from no review of research scans and limited feedback to research participants, to routine review of scans and the arrangement of clinical follow-up. Respondents felt that researchers do not have a duty to actively look for incidental findings, but they do have a duty to act on findings, when detected. The principle of reciprocity featured prominently in our interviews and expert meeting.

**Conclusion:**

We present an ethical framework that may guide researchers and research ethics committees in the design and/or evaluation of appropriate pathways for the handling of incidental findings in imaging studies. The framework consists of seven steps: anticipation of findings, information provision and informed consent, scan acquisition, review of scans, consultation on detected abnormalities, communication of the finding, and further clinical management and follow-up of the research participant. Each of these steps represents a key decision to be made by researchers, which should be justified not only with reference to costs and/or logistical considerations, but also with reference to researchers’ moral obligations and the principle of reciprocity.

## Background

Research groups worldwide are studying the human body making use of non-invasive imaging techniques, notably magnetic resonance imaging (MRI). On MR images, researchers will incidentally come across so-called incidental findings, findings that are beyond the aims of the study but may be of potential health or reproductive relevance to - otherwise healthy - research participants [1]. Detection and feedback of incidental findings are a double edged sword: they may allow for timely treatment and thus lead to medical benefit, but may also harm research participants because of the risks, psychological burdens and costs of follow-up testing and (over)treatment [2, 3]. Not all abnormalities warrant clinical follow-up, [4–6] or lead to the possibility of procuring health benefits [4]. Thus, incidental findings tend to place researchers before an ethical dilemma: to actively search for these or not, and to refer for further work-up or to remain silent.

The prevalence of clinically significant incidental findings among healthy participants is estimated around 2.7% in brain MRI, with a number needed to scan of around 37 for one finding deemed of clinical significance [7]. Though the prevalence of incidental findings may be lower in children and adolescents than in the elderly, they are not extremely rare in the young [8, 9]. In imaging of other body parts (e.g. abdomen or whole-body), the frequency with which incidental findings are detected may be much higher [10, 11]. In Europe, large population-based cohort studies are currently underway, in which (tens of) thousands of healthy research participants are undergoing imaging of the body or brain – in the case of UK Biobank, even a hundred thousand [10, 12–16]. Large numbers of incidental findings can thus be expected in the next few years.

International guidelines for the detection, management and feedback of incidental findings in the context of research have, as of yet, been largely procedural and quite non-specific, and have allowed for a “range of options” [17] or a “spectrum of models” [18] to evolve for the handling of incidental findings [19, 20]. For instance, whereas in some studies, research scans are checked for image quality only and very limited feedback is given to research participants, [13] other groups conduct a full clinical review of all research scans by expert radiologists [10]. Practices for the handling of incidental findings thus vary considerably across studies and research settings [20, 21].

Although current guidelines are non-specific in how incidental findings should be handled, they do agree on some important points. When abnormalities of potential clinical significance are being picked up by researchers, they should act [1, 19, 22, 23]. Further, researchers ought to consult with an expert (usually a radiologist) to confirm the imaging finding before informing the research participant [20, 24]. Moreover, a predesigned pathway for the handling of incidental findings, including expert consultation, should be in place and should be communicated beforehand with research participants, preferably as part of the informed consent process [1, 19, 22, 25]. Finally, the pathway should ideally be reviewed by an ethics committee or institutional review board [26]. However, as general ethical frameworks are still lacking, [25] ethics committees may currently be insufficiently equipped to evaluate pathways for the handling of incidental findings within research protocols.

With the rise of the use of imaging techniques in research comes an urgent need for practical ethical guidance. Researchers need to know what adequate pathways for the handling of incidental findings look like. In this paper, we present a seven steps framework to be used by researchers and/or ethics review committees when setting up or evaluating pathways for the handling of incidental findings in imaging studies. Researchers will need to consider each of the seven steps of the framework, will need to decide how to give them shape in a pathway for the handling of incidental findings, and will need to justify these decisions, taking research participants’ moral expectations into account. The seven-step framework has been developed based on an interview study and an expert meeting on current practices for the handling of incidental findings.

## Methods

This paper explores current practices for the detection, management and communication of incidental findings in neuroimaging and other research centres in the Netherlands, as well as - senior and junior - researchers’ perspectives on their moral obligations with regard to incidental findings. The approach taken consists of two components: an interview study and an expert meeting.

As part of an extensive qualitative research project of stakeholders’ perspectives on incidental findings, we conducted semi-structured individual and group interviews with researchers, researchers-radiologists, and research managers involved in imaging studies or biobanking at seven Dutch research centres or academic institutions (total *n* = 20), including both hospital-based or hospital-affiliated research centres and dedicated neuroscience research centres. Respondents were involved in brain imaging (MRI) for various research aims, medical and non-medical, either as stand-alone imaging studies or as part of wider sets of tests and examinations within larger studies. PhD-students from various institutions were also interviewed to compare daily practice with institutional policies. Further, general practitioners who are involved in imaging studies, a medical psychologist, directors of screening programmes and a director of a biobank (see Table 1), about their views on and experiences with incidental findings. All respondents were interviewed at their work locations in various regions across the Netherlands. At two research centres (both population-based cohort studies), the researchers (LvB and EB) were invited to observe the scan acquisition processes, and conducted informal interviews with research assistants and radiographers. Also at 4 centres (two population-based cohort studies, neuroimaging centre A, hospital-based imaging centre), respondents made available participant information sheets, informed consent forms, and/or internal documents outlining policies for the handling of incidental findings to the researchers. At other centres, respondents did not demonstrate the research process or make available additional materials to the researchers. Interviews lasted between 60-90 minutes, and were carried out by one (LvB) or two (LvB and EB) researchers between March and May 2014. Respondents were asked to explain their institutional pathways for the handling of incidental findings, to identify ethical issues, and to elaborate on their views on researchers’ moral obligations with regard to incidental findings.

**Table 1:** Respondents included in the interview study

Respondent	(Bio)medical discipline	Position	Type of research centre
1	Neuroradiology	PI	Population-based cohort study A
2	Neuroradiology	PhD-student	Population-based cohort study A
3	Neuroradiology	PI	Population-based cohort study B
4	Neuroradiology	PI	Neuroimaging centre A
5	Neuroscience	PhD-student	Neuroimaging centre A
6	Neuroscience	PhD-student	Neuroimaging centre A
7	General medicine	PhD-student	Hospital-based imaging centre
8	Psychiatry	PhD-student	Hospital-based imaging centre
9	Neuroscience	PI	Neuroimaing centre B
10	N.A.	Research manager	Neuromaging centre B
11	General medicine	PhD-student	Neuroimaging centre B
12	Neuroscience	Research assistant	Neuroimaging centre B
13	Radiology	Director	National screening programme A
14	N.A.	Director	National screening programme B
15	N.A.	Legal advisor	National screening programme B
16	General medicine	Medical advisor	National screening programme B
17	Pathology	Director	Biobank at academic hospital
18	Medical psychology	Psychologist	Academic hospital
19	Primary care	General practitioner	Private practice
20	Primary care	General practitioner	Private practice

The interviews were digitally recorded, transcribed verbatim and stored securely. Emotional responses (e.g. ‘sigh’ or ‘silence’) were also noted by the interviewer(s). This was done as deemed relevant by the interviewer, not done in any systematic way. Paper-based and basic word processing methods were used for data analysis. Interview transcripts were coded independently by two researchers (LvB and EB) using a constant comparative method, [27] which entails going back and forth between transcripts and codes and themes in an ongoing, iterative process [28]. Codes were assigned, regrouped into higher-order themes, and refined through multiple readings and re-readings. Discrepancies were resolved through discussion. Main themes were identified, and were used to collect and regroup transcript fragments.

Based on the results of our interview study, we have drawn up an ethical framework for the detection, management and communication of incidental findings detected through imaging in research, consisting of seven points that should be considered prior to the start of every imaging study (see Discussion). This framework was presented and corroborated at a 2-day international expert meeting, organised at the Department of Medical Ethics and Philosophy of Medicine in Rotterdam in 2015, in which representatives were gathered from large European population-based cohort studies as well as from various research centres in the Netherlands. The attending experts (*n* = 14) came from the Netherlands, the United Kingdom, Germany, Sweden and Belgium. Apart from (neuro)radiologists and (neuro)science researchers, the group also involved ethical and legal experts. Some attendants were not involved in large population-based studies, but in neuroscience or genomics studies. The main aims of the expert meeting were to share best practices and moral considerations, and to discuss our ethical framework for the handling of incidental findings in research. Based on the expert meeting, we fine-tuned, complemented and adjusted our framework. The outcomes of the expert meeting will be presented in the discussion.

## Results 

First, we give an impression of the range of pathways that currently exist in the Netherlands for the handling of incidental findings. Then we outline how and why our respondents made choices about the set-up of their adopted pathways. Finally, we report on our respondents’ views on researchers’ moral obligations.

### Pathways and policies for the handling of incidental findings differ across the Netherlands

Pathways and policies for the detection, management and communication of incidental findings were variable: some centres had detailed pathways in place, outlining what to do and whom to contact in case abnormalities were detected, while other centres were still in the process of setting up policies or pathways for the handling of incidental findings, or were struggling with incidental findings on a case-by-case basis. While most respondents supported policies that ensured that potentially relevant incidental findings are reported back to research participants, others maintained limited or ‘no feedback’ policies. In four centres, internal documents described standard operating procedures researchers or radiographers were expected to follow when confronted with abnormalities. While these procedures were largely similar (e.g. scans should be saved in specific ways and on specific locations, a person centrally handling incidental findings should be contacted, the researcher should wait for a confirmatory response from a (neuro)radiologist), there were also differences with regard to whether or not researchers or radiographers were required to make additional diagnostic-quality scans of potentially abnormal brains, and whether or not they were allowed to inform the research participant about the finding during the scanning process.

Moreover, young researchers or radiographers were given different, sometimes conflicting instructions: in some centres, researchers were actively discouraged from ‘looking for’ abnormalities on scans, while in other centres, researchers were trained ‘how to check’ for incidental findings. These differences are illustrated by the following fragments from a group interview with three PhD-students working at two imaging centres in the same city:

- *PhD-student a: You’re not really going to look for things, right? […] But if you really see something, you report that. That’s what the protocol says.*
- *PhD-student b: But isn’t there a hospital-broad protocol that says: ok, this is how you check for incidental findings?*
- *PhD-student c: No, you don’t really have to check, right? It is an incidental finding when you come across something.*
- *PhD-student a: No, that’s right, there is no need to check. But if you see something, you report. That’s the protocol. But it’s not like everyone will look at scans in the same way or search for things…*
- *PhD-student c: I think it’s right not to look for things.*

Policies with regard to the feedback of imaging results to research participants varied, as well. In some centres, researchers were instructed to refrain from providing any information to research participants, while in other centres, researchers were encouraged to offer research participants their scans on CD.

- *PhD-student a: So you make a screenshot?*
- *PhD-student c: Only if [participants] ask. We don’t routinely offer it to them.*
- *PhD-student b: You don’t routinely offer it to them? We always just give it to them.*
- *PhD-student c: What? A screenshot or a CD?*
- *PhD-student b: A CD with everything […]*
- *PhD-student a: So you have all the images to scroll through?*
- *PhD-student b: Yes […] Does your centre really advise against that?*
- *PhD-student c: Definitely. I am told not to even show the computer screen to participants […]*
- *PhD-student b: They really like it […] Especially since it is about their own brain […] One participant had even printed an image on his T-shirt!*
- *(PhD-students a and c (respondents 7 and 8) work at a hospital-based imaging centre, PhD-student b (respondent 5) works at an independently located neuroimaging centre.)*

### Pathways: seven steps

After learning from all of our respondents about various existing pathways for the detection, management and communication of incidental findings used in several research centres across the Netherlands, we identified seven crucial steps in these pathways: anticipation of findings, information provision and informed consent, scan acquisition, review of scans, consultation on detected abnormalities, communication of the finding, and further clinical management and follow-up of the research participant. Together these seven steps form an ethical framework for the handling of incidental findings. Below, the findings from our interview study will be presented along the lines of this seven step framework. In each of these seven steps, researchers have to make choices with regard to the pathway for the handling of incidental findings, and have to justify these choices. These choices and their justifications were the topic of many of our discussions with respondents.

### Anticipation of incidental findings

Not all research centres have prepared for the occurrence of incidental findings to the same extent. This may be because incidental findings tend to occur more frequently in clinical or population-based cohort studies than in neuroscience, cognitive science or behavioural science studies, and are perceived to pose more of a problem in the former than in the latter:

*We’ve been here for twelve years by now, we have [acquired brain scans from] 3,000 people annually. There have been [a few] incidental findings, but no one has died from them. […] The panicky idea that we could be saving lives by detecting incidental findings, I am not buying that. (research manager at neuroimaging centre b, respondent 10)*

*So there is a big problem, I can tell you that. As a doctor you cannot lay awake at night […] But no one should deny that this is a big problem. (neuroradiologist at population-based cohort study a, respondent 1)*

The differences in frequency – and concurrently in problem perception – may be explained in part by differences in age ranges of the scanned participants: in some neuroscience centres, research participants are typically young students seeking to acquire university credits or to earn some extra money. Few clinically relevant incidental findings seem to be detected in healthy young volunteers:

*Really, we’re talking about one case in five years, or something. […] That is what the fuss is all about. Everyone is really focused on this. It is different with the elderly. Maybe you should [discuss incidental findings] with the elderly, but not with the young. (principal investigator (PI) at neuroimaging centre a, respondent 4)*

Some centres, in which incidental findings occur with a relatively high frequency, have explicit protocols in place for their management. At one centre, an interdisciplinary expert panel has assessed the practical and clinical utility of relatively frequently occurring incidental findings and compiled a list of findings to report and findings not to report to research participants. Rare, unexpected incidental findings that have not been anticipated in the protocol, are discussed during a panel meeting to decide whether or not to notify the research participant:

*We have really made a list that explicates which findings we report and which findings we intentionally do not report. And everything in between, everything else we encounter, we will have to discuss anew. (neuroradiologist population-based cohort study b, respondent 3)*

At other centres, where policies have not been written down, respondents did have a sense of the criteria that are used, implicitly or explicitly, in discussions about what incidental findings to report and what findings not to report. Respondents mentioned criteria such as a serious or life-threatening disease, treatability or ‘actionability’ (clinical utility), or whether informed consent was given to learn about incidental findings:

*I always just stick to this, together with the rest of the [hospital], that it should be life-threatening and treatable before you are going to burden someone with [an incidental finding]. (director of a biobank, respondent 17)*

### Information and informed consent

Many respondents felt it is important to inform research participants beforehand about the possibility that incidental findings may be detected during research. Some respondents however felt no need to explicitly discuss the issue of incidental findings beforehand, as they occur only rarely or have little clinical importance. Further, researchers worried that information about incidental findings would cause (needless) anxiety or apprehension in research participants, so that they may no longer be willing to participate in research:

*Incidental findings are of such little relevance that you don’t even need to discuss them with subjects. You don’t even need to talk about incidental findings in the informed consent. Because they have no consequences in the young. (PI at neuroimaging centre a, respondent 4)*

*I am wondering whether [pre-scan information on incidental findings] may cause more harm than good, because you may deter [research participants] more, so that they may no longer be willing to participate. And they may overestimate the chance that something will be found. (PhD-student a at neuroimaging centre b, respondent 11)*

At most centres, however, researchers are obliged by internal policies to inform research participants that incidental findings may be detected. As part of the informed consent, research participants will have to agree to receiving feedback about any clinically relevant incidental finding. Often, participants will also have to agree to the researchers notifying their primary care physician in case an abnormality is detected.

During the informed consent process, research participants are often told that research scans are not diagnostic scans, that research scans will not be reviewed by medical doctors, that incidental findings are rare, and that not hearing back from the researcher does not signify that the research participant is healthy or free from abnormalities. Some respondents felt uncertain whether research participants understand the information provided, or felt that the information itself is ambiguous:

*Look, you can say: ‘This is scientific research, no medical doctor is going to be looking at [these scans], no clinical eye, there is no such thing as gaining medical benefit from this’. But then you’re also saying: ‘If we do find something very odd, we will tell you.’ Well, then you’re dithering: we’re not looking, but we’re looking. This is an ambivalent message, if you ask me. (primary care physician a, respondent 19)*

*Well, I think it is important to explain [that you are not looking] to research participants, but I do think that people will think, in the back of their minds: ‘Oh, but if there really is something wrong, those [researchers] will see that, and then they will send [the scan] on [to an expert radiologist]. I do think that [scanning] offers false reassurance. (PhD student at a hospital-based imaging centre, respondent 8)*

Despite serious attempts to downplay participants’ expectations with regard to clinical review during the informed consent process, respondents felt that many research participants continue to assume that apparent abnormalities will be detected and acted upon by researchers.

### Scan acquisition

Typically, in clinical or population-based cohort studies, the sequences acquired (T2 weighted or fluid-attenuated inversion recovery (FLAIR) images) are of higher diagnostic utility than the sequences acquired (T1 weighted or functional MR images) in more fundamental research, e.g. neuroscience studies. Diagnostic-quality images will generally lead to the detection of more incidental findings than functional MR images. But even in research centres that are not hospital-based, in which only functional MR images are acquired in the context of studies that have non-medical aims, the possibility of detecting abnormalities is often brought up by volunteers. Sometimes, researchers are psychology or neuroscience PhD-students with limited medical background and may not be able to confidently answer volunteers’ questions:

*It is an often recurring issue, in discussions with participants, too. […] They will always be asking: ‘Well? Does everything look normal?’ (PhD-student at neuroimaging centre a, respondent 6)*

At other research centres, radiographers are operating the MRI scanner, and researchers will not have direct contact with research participants. Both radiographers and PhD-students seem well aware that they are not in a position to offer medical/clinical feedback to research participants during or following a scan. Respondents felt that radiographers or researchers are under no (formal) obligation to check for abnormalities during scan acquisition:

*The researchers perform the scanning themselves. They will have to become certified users, but that has only to do with technical skills and not with the assessment of the brain at an anatomic level. So these are not people who know what a brain looks like. So they either detect something by coincidence or they don’t. There is no obligation on the part of the researcher to scroll through every anatomical scan, slice by slice, to see is there is something wrong. (PI at neuroimaging centre b, respondent 9)*

Some researchers feel that they should (briefly) check research scans for abnormalities anyway, while operating the scanner, whereas others do not. Research scans are thus ‘looked at’ differently by different researchers.

### Review of scans

The extent to which research centres review brain scans varies. Some centres ask researchers or radiographers to assess the quality of the scan only, on the spot, during scan acquisition: have the required sequences been made, are all required measurements or biomarkers present and clearly visible? In those centres, no arrangements have been made for the review of research scans for abnormalities, and PIs may tell junior researchers or radiographers that the detection of incidental findings is something to be avoided. These are usually centres in which only functional imaging sequences with limited structural sequences (only T1-weighted) are acquired, the diagnostic utility of which is limited. Such images do not easily allow for the adequate detection or clinical interpretation of certain incidental findings, such as aneurysms, although large tumours may be spotted.

Other centres have set up a system in which all diagnostic-quality research scans are reviewed for abnormalities, either directly by an expert neuroradiologist or indirectly, after possible incidental findings have been flagged by research personnel. One centre especially trains their PhD-students (most of them have a medical background and are training to become radiologists), through a training programme that was developed in-house, to distinguish normal brains from abnormal brains. When the PhD-students detect an abnormality, they put it on the agenda of a two-weekly team discussion, led by an expert neuroradiologist. The expert neuroradiologist will explain the abnormality to the researchers and decide whether or not to report the finding to the research participant.

One respondent from another centre in which routine clinical review of all research scans is meant to be conducted, explains that the centre lacks the manpower and the financial means to review scans in a timely manner. The respondent is not comfortable with this situation, as it entails that research participants will need to be notified of an incidental finding a long time after the scan was acquired. Research participants may not understand or appreciate the time lag, especially when children are involved as research subjects:

*This is a seven-year-old child. The scans had not been reviewed before. Because, well, that is because of a backlog in reviewing. Because, you know, it is a lot of work. And it is work that is unpaid. No reimbursement. A year later we are analysing these images for a scientific research question, and the researcher is saying: ‘Hey, something is wrong here’. We’re seeing this asymmetry [possibly a tumour]. (neuroradiologist at population-based cohort study a, respondent 1)*

### Consultation on detected abnormalities

Many centres instruct researchers that when they do detect an abnormality, they must contact the principle investigator or manager of the research centre. The PI or the manager may not be an expert (neuro)radiologist him- or herself, but he or she *is* in a position to decide whether or not consultation by an expert will be necessary. It is generally agreed upon that incidental findings should be confirmed by an expert (usually a radiologist) before the research participant is informed.

One respondent was concerned with the potential harms and burdens of asking for expert opinion. This respondent felt that as research participants are presumed healthy and free of symptoms, incidental findings will – almost by definition – have limited clinical significance and will not affect the participant’s daily life in any way. Incidental findings, in this respondent’s opinion, will not lead to medical benefit, and may only cause harm:

*With [referring the research participant for expert consultation] you will needlessly trouble the research participant. If it may not affect the participant’s daily life, you will have to ask yourself: are you really going to start an entire procedure, in which you’re asking the neurologist and the primary care physician for their opinion? This will only upset the research participant, often for no reason. (primary care physician b, respondent 20)*

It can be difficult to find expert (neuro)radiologists who are willing to assess incidental findings on a case-by-case basis, even if they were to be reimbursed. One respondent from a neuroimaging centre indicated this. Radiologists, our respondent thought, are hesitant to take on the liability of reviewing research scans, and fear that they would miss something:

*Well, we choose not to have radiologists [routinely] look at scans. That is too expensive. We don’t do that. So it becomes an incidental finding. […] Then you need someone [an expert radiologist] who can assess whether it is [an abnormality that needs reporting] or not. Well, you go and try to find such a person. (research manager at neuroimaging centre b, respondent 10)*

### Communication of the incidental finding

Respondents felt that the communication of an incidental finding should be undertaken by medical doctors. Researchers who are not doctors, some of our respondents said, cannot legally enter into a physician-patient relationship, in which a diagnosis can be conveyed or recommendations for tests or treatments can be given. Research centres will thus rely on in-house medical doctors to tell the participant about the incidental finding, or need to refer the participant to an external doctor for the feedback of the finding. One respondent said that for his centre, it is not practically or financially feasible to hire doctors to communicate findings. Therefore, this centre will not provide feedback on any findings at all.

*We cannot afford feeding back this information. […] I cannot do it myself […] because I am not medically trained. […] So a physician would have to be arranged to feed the information back. And for those reasons we have decided: we’re not going to do it. (director of a biobank, respondent 17)*

Some respondents feel that incidental findings should be communicated via primary care physicians. It is an internal policy in many research centres that researchers contact the participant’s primary care physician after the finding has been confirmed. Informed consent forms usually contain the name and contact details of participants’ primary care physicians. Researchers will send a letter to the primary care physician, explaining the finding and include the expert report. The primary care physician, it is thought, will be able to contextualise the finding and decide whether or not to tell the research participant:

*I strongly support that [IFs are reported through primary care physicians]. The primary care physician knows the social situation of the patient, knows the co-morbidity, has a better understanding of [the patient…] I think the primary care physician is the right person. Just to take the context into account when communicating with the patient, but also for the follow-up. (primary care physician a, respondent 19)*

One respondent disagreed. Research participants may feel bypassed when researchers would contact their primary care physicians without them knowing. As a researcher and medical doctor, this respondent usually contacts research participants directly, from the research centre, over the phone, to tell them about an incidental finding and to refer them to a clinical specialist (i.e. neurologist, neurosurgeon):

*We offer participants the chance to choose against acting upon an incidental finding. So that we do not send them into the medical ‘circus’ against their wishes. So we always ask the question [whether or not they want to follow up on the finding]. We do not seek contact with the primary care physician before talking to the participant him- or herself, because that feels like sharing private information behind someone’s back. The participant him- or herself should control that information. So on the phone I always ask: ‘Now, I have told you this, and probably your primary care physician would like to know about this, too. Are you OK with that?’ (neuroradiologist at population-based cohort study b, respondent 3)*

In practice, very few research participants keep the researcher from letting their primary care physician know about the finding.

### Further clinical management and follow-up

In some cases, hospital-based or hospital-affiliated respondents have made prior arrangements or maintain relationships with clinical specialists, so that whenever they notify a research participant about an incidental finding, an appointment with a clinical specialist can be arranged within a timeframe of a couple of days. This approach, it is thought, will reduce uncertainty and anxiety in research participants. Other respondents indicated that they simply refer research participants to their primary care physicians, who may then arrange a meeting with a clinical specialist for them, if deemed necessary.

Because researchers are not in a physician-patient relationship with their research participants, they rarely hear back from them, and often do not know what happens to research participants after they have been told about incidental findings.

*What I have heard […] is that it all went well and that, given the circumstances, the information [about the incidental finding] has been received well. So that is what we hear back. But because we are not in the role of the physician, we don’t get to hear any details. So you will just have to assume that it went well. (director of screening programme a, respondent 13)*

*We have had that question from the ethics review committee: ‘Can you not follow up on these research participants?’ But when they are in the care of a physician, [the physician] will have his professional confidentiality, and we are not medical doctors. We are not a medical centre, so a doctor can tell us nothing, unless the research participant indicates that he wants his doctor to inform us, but the research participant will have other things on his mind. (PI at neuroimaging centre b, respondent 9)*

One respondent did follow up on incidental findings as part of a research protocol, and gathered information from medical specialists on the tests and treatments offered to research participants. This information was used to adapt the protocol for the handling of incidental findings:

*At one point we had referred thirty people or so to the endocrinologist [for small pituitary cysts]. And then there were hormone examinations and new scans. After that we drew up the balance sheet and it turned out that none of these thirty people were suffering from hormone disorders. […] So we decided in a team conference that it was probably not that useful to refer people with such an abnormality. (neuroradiologist at population-based cohort study b, respondent 3)*

### Moral responsibilities

During our interviews, we sought to elucidate researchers’, research managers’ and other stakeholders’ views of the moral responsibilities of researchers. Do they think researchers are morally required to ‘look for’ incidental findings? Do they think they are to be blamed when they ‘miss’ incidental findings? What are researchers’ moral considerations regarding the detection, management and communication of incidental findings? What do they perceive to be their (implicit) mission statements as researchers?

### Researchers’ responsibilities to look for incidental findings

Many of our respondents did not consider it a researcher’s responsibility to actively look for incidental findings. Researchers should focus on conducting research and need not employ a ‘clinical eye’ when looking at images or other research data. Correspondingly, there is no such thing as a moral or legal liability for failing to notice or missing an incidental finding:

*You have to think about what the responsibilities are of someone who is holding the materials and looking at the results of those tests. This [researcher] is not trained to find such things. So if he is alert, he’ll notice, but you can’t blame him if he’s not alert. (director of a biobank, respondent 17)*

Several researchers remembered cases in which the research centre had been approached by research participants or their family members, when they fell ill. Researcher participants or their family members had demanded clarification: Had the condition not been visible on the research scan that was made at the centre a few months before? Sometimes the researchers had to admit that the abnormality was indeed visible on the scan, but had not been noticed by the researchers. At other times the researchers concluded that the abnormality had not been present at the time of the research scans. Either way, although such cases seemed to have made an emotional impression on researchers, most did not think they were culpable.

*This woman [whose mother recently died] came to the clinic to see the neurosurgeon and tells the neurosurgeon: ‘My mother participated in a scan study 8 months ago. Have they not seen anything?’ So the neurosurgeon calls the researcher and asks: ‘Have you not seen the tumour?’ So we go and look at the scans […] Here you see a small aberration. These images had been reviewed. [The aberration was] not detected. So we have to tell this patient: ‘Yes, we checked the images again, and in retrospect we can see something. […] Yes, we can see it now, but we missed it then […] And now she died. (neuroradiologist at population-based cohort study a, respondent 1)*

*We call it the retroscope: when you know where something has been found, when you look back exactly in that place, you increase your sensitivity to find something there. Then we said: if we’re looking at both scans, we can now see that something was amiss on that first scan, but if we had only the first scan […] we couldn’t have seen it then. It is not like we missed something. (neuroradiologist at population-based cohort study b, respondent 3)*

### Researchers’ responsibilities to report incidental findings

The respondents thought that it would be wrong not to tell research participants about clinically relevant incidental findings, when detected. Some incidental findings may not cause symptoms now, but they may start to do so in the future. When research participants and their doctors have been told about the finding, they may be spared unnecessary diagnostic delays, and the burdens and costs associated with such delays.

*You cannot do that: keeping things from people. Not the things which are relevant enough to report to a primary care physician. (primary care physician b, respondent 20)*

*Think about this: at a certain point in time this person develops a headache or something, and we all know how long it takes for a patient to go through the medical parade before [doctors] order a brain scan, and it turns out to be a cyst. And we would have known all along [that it was a cyst…] I do understand that you shouldn’t worry people unnecessarily, but on the other hand I do think it would be strange to withhold this information from a research participant. (neuroradiologist at population-based cohort study a, respondent 1)*

One respondent disagreed, and felt that researchers have a responsibility to decide carefully whether or not it will benefit the research participant to receive information about the incidental finding. In many cases, incidental findings will prove of limited clinical utility, this respondent thought, and will only harm research participants. The researcher should thus decide not to tell the research participant:

*The moral obligation, I think, is also to build some form of protection against those five dots on the MRI that mean nothing but could haunt people for the rest of their lives. (director of a biobank, respondent 17)*

### Researchers’ care obligations

Respondents were well aware of the differences between the legal and moral obligations of physicians caring for patients on the one hand, and researchers conducting research with the help of research participants on the other hand. Still, researchers felt that there are special moral obligations that flow from the researcher-research participant relationship. Researchers cannot treat research participants like instruments or models, but show - or should show – concern for research participants.

*I believe [it has to do with] integrity. Like: this is how we deal with our research participants. We’re not just hauling them in and out of our scanners, and that’s it, you get paid or you get [university] credits [in case of students]. We take a broader perspective. This is not about care per se, but it is about, well, concern. We’re not in a care relationship – these are not patients. They are research participants, for whom you do take responsibility. (PI at neuroimaging centre a, respondent 4)*

One respondent felt that researchers, like doctors, are responsible for the health of their participant. Research participants expect researchers to care for them. This may apply even more to researchers who are also physicians.

*[Research participants] expect you to feel responsible. You can try to demarcate and say: ‘Well, this is research, we are only looking at the science, we’re not looking after your health, but that is not how life works. People expect you to [look after their health]. There are expectations. (neuroradiologist at population-based cohort study a, respondent 1)*

### The principle of reciprocity

The concept of expectations was often brought up during our interviews. Some of our respondents felt that they were expected to look after research participants’ health, to look carefully at research data, to check whether ‘everything looks normal’. To these respondents it seemed that one of the main motivations for volunteers to participate in studies is to receive a ‘health check’. These respondents mentioned the principle of reciprocity, which would suggest that research participants’ expectations (i.e. the health check) should be met, and that indeed, researchers should check images for abnormalities.

*I think it’s good [if researchers feed results back], because [research participants] value that. I think it’s a type of exchange: you participate and you want an answer, then you shall naturally have that answer. [It is] a social interaction. (neuroradiologist at population-based cohort a, respondent 1)*

*We know that most people participate because they see this as a [free medical] check-up. (primary care physician a, respondent 19)*

*And you notice […] that many people participate in research, secretly with the idea that they will learn about serious abnormalities in the brain. (PI at neuroimaging centre b, respondent 9)*

Other respondents chose to clearly inform research participants beforehand that the imaging study does not function as a health check, and will not lead to medical benefit for participants.

## Discussion

Existent ethical and legal guidance, so far, has suggested that a pathway for handling incidental findings should be in place at the outset of the research program, evaluated by an ethics committee and communicated to research participants as part of the informed consent process [1, 26]. However, no specific criteria have been established for the setting up of such a pathway or for the evaluation of pathways within study protocols. Is there really nothing more to say – normatively – about pathways for handling incidental findings, over and above that they should be present and should be evaluated on a case-by-case basis? Based on our interview study and expert meeting, we contend that there *is*: any responsible pathway in a high-income country must, at minimum, adequately address the seven steps listed in our framework. It should be noted that, as this study was conducted in the Netherlands, the outcomes and the resulting ethical framework cannot be translated directly to other countries, such as low and middle-income countries.

The seven steps of the framework need not and cannot be addressed in precisely the same manner in all research studies. Given the reported variability between research settings, it will hardly be possible to converge on a single ethical standard for the design of pathways for the handling of incidental findings in (brain) imaging research [23, 29]. Highly demanding pathways may not be feasible or desirable across all research settings, e.g. in low-resource countries. Moreover, in research settings in which only functional brain imaging is acquired for non-medical purposes, the chances of detecting interpretable, clinically relevant incidental findings are slim. It may be unnecessarily burdensome to trouble volunteers with information on the possibility – however small – of incidental findings and the implications thereof. Ethical guidance will thus have to be differentiated [29].

The seven steps framework therefore, cannot serve as a cookbook, but rather points out a series of decisions that each research team will have to make and will have to justify. Decisions should be justified in the light of not only considerations of feasibility, logistics and cost [23, 25], but also medical-ethical considerations such as potential harms and benefits or the principle of beneficence, [18] and respect for research participants [30]. Researchers and ethics committees will need to balance these considerations on a case-by-case basis and make trade-offs between the financial and organizational costs of a pathway and its potential for medical benefit. However, especially in high-income countries, a set of minimum requirements should be met (see Table 2). The costs need not be prohibitive: it has been found that a ‘universal review and disclosure system’ can cost as little as 23 US dollars per scan [31]. Even limited potential medical benefit – or the principle of reciprocity - might outweigh such costs in high-income countries. On the other hand, researchers should protect research participants against unnecessary harm [32], and need not inform them about clinically insignificant – and potentially harmful - incidental findings. After all, the reporting of incidental findings can be in the interest of research participants, but may also have adverse implications [2].

**Table 2:** The seven steps framework and minimum requirements for pathways for the detection, management and communication of incidental findings in research

The seven steps framework		Minimum requirements
1.	Anticipation of incidental findings	Incidental findings should be anticipated.
		In large-scale imaging studies, lists should be made of anticipated incidental findings, stipulating their management.
2.	Information provision and informed consent	As part of the informed consent process, research participants should be informed about the possibility that incidental findings may be detected, and about the pathway for handling such findings.
		Research participants should be given the opportunity either to opt out of receiving information about incidental findings or to withdraw from the study.
3.	Scan acquisition	Researchers need not acquire any (diagnostic-quality) scans in addition to the scans that are necessary for the research purposes.
		Radiographers should be instructed whether and to what extent to review scans for abnormalities during scan acquisition
4.	Review of scans	In studies in which diagnostic-quality images are acquired, some form of routine review of research scans should be arranged.
5.	Consultation on detected abnormalities	Detected abnormalities should be confirmed by experts (i.e. radiologists) before they are reported to the research participants.
		Researchers should make prior arrangements with experts.
6.	Communication of the incidental finding	Policies for the communication of the incidental finding to the research participant should align with national regulations and customs.
7.	Further clinical management and follow-up of the research participant	In case of serious incidental findings, researchers should take responsibility for the clinical follow-up of the research participant (i.e. through adequate and timely referral).
		Researchers should make prior arrangements with relevant clinicians.

Our respondents recognised that researchers are held by special moral responsibilities with regard to the detection and careful handling of incidental findings. This is consistent with the entrustment model of the researcher-research participant relationship [33, 34], which sets out role-specific ancillary care obligations for researchers. These obligations follow from the informed consent process, through which research participants entrust aspects of their health to the researcher. Based on this ‘partial entrustment’, researchers have a duty of rescue towards research participants that is stronger than that of ordinary citizens towards one another [34, 35], and are expected to care for entrusted aspects of research participants’ health (e.g. the brain). Researchers would thus have a strong role-specific moral imperative to act upon clinically relevant incidental findings detected during imaging.

In addition, the principle of reciprocity featured prominently in our interviews with researchers (and in our interviews and focus groups with research participants, which will be reported separately), and gained traction during our expert meeting. Our respondents believed that an adequate response to incidental findings is an expression of respect for research volunteers’ contribution to scientific research. Studies have shown that many research participants expect that incidental findings, if present, will be detected and will be acted upon [25], and have a strong preference for receiving information about incidental findings [36–38]. Based on the principles of respect for (the autonomy of) research participants and of reciprocity, researchers may have reasons to check for abnormalities when they can.

Ethics review committees will have an important part to play in shaping researchers’ responsibilities, by evaluating and refining pathways for the handling of incidental findings within research protocols. The seven steps framework can help guide ethics committees’ deliberations. In addition, we will now provide some considerations to help determine the adequacy of researchers’ decisions with regard to the management of incidental findings. These considerations reflect current ethical guidance and best practice, and although such high standards may not be achievable for all research groups at present, they do set an example. Furthermore, based on ethical discussions in the literature, our interview study and expert meeting, we outline some minimum requirements for each of the seven steps (see Table 2).

### Anticipation of findings

There is an increasing body of literature showing that incidental findings can and should be anticipated [10, 19, 26, 39]. Researchers who are involved in setting up an imaging study should acknowledge that incidental findings may occur. Researchers should assess the type and the frequency of findings to be expected, based on the type of scans that will be acquired and the study population. For large-scale studies in which diagnostic-quality scans are acquired, it is considered best practice to determine, prior to the start of the study, which (types of) findings, if detected, will or will not be reported. Representatives of large population-based studies who attended our expert meeting recounted that their respective research groups had agreed upon a list of findings to be reported and a list of findings not to be reported. They had established multidisciplinary panels, in which radiologists and clinicians worked together to develop such lists. Some studies have used the Rotterdam Study protocol for the handling of incidental findings [40], which contains a list for brain MRI, as a model. In drawing up a list of anticipated incidental findings to be reported, the clinical validity and utility of the findings [23] should be central criteria. This does not imply that information should only be returned if it pertains to diseases that can effectively be treated.

There are limits to the utility of lists: not all incidental findings can or need be anticipated. Attendants at our expert meeting reported that incidental findings that are *not* anticipated - and therefore not included in lists - are discussed on a case-by-case basis in multidisciplinary panels. Also, such panels serve to evaluate and revise the lists over time, as experience with incidental findings accumulates within studies.

### Information provision and informed consent

It is generally agreed upon that as part of the informed consent process, research participants should be informed about the possibility that incidental findings can be detected and about the study’s pathway for the handling of incidental findings [26], including when and how research participants will be informed about an incidental finding, if detected. Ideally, research participants should be given the opportunity to consent to research participation while opting out of receiving information about incidental findings [19, 35, 36, 41]. Allowing participants to opt out helps to avoid unnecessary pressure to learn about - possibly unwanted - health-related information. Researchers or ethics committees may wish to qualify such an opt-out option, so that, if feedback of an incidental finding were necessary to prevent serious harm to the research participant [42], an exception could be made and the finding could be reported anyway. This option is called a ‘conditional opt-out’ option. Experts who attended our meeting indicated that generally only a small minority of research participants chooses to opt out of receiving information about incidental findings, and reported that it should be feasible to check the informed consent form first for research participants’ stated preferences, before contacting them.

### Scan acquisition

Researchers must inevitably decide in advance which sequences will be acquired during the study. Current guidance suggests that researchers are not required to make any additional scans for the purposes of increasing the likelihood of detecting incidental findings [26]. Thus, the scanning protocols should simply be the minimum required to answer the research question [22]. This implies that researchers can perfectly well acquire scans of (very) limited diagnostic utility only. This was corroborated in our expert meeting. Further, radiographers or researchers who are performing the scans should be instructed whether or not to review scans during scan acquisition, and to what degree, for the presence of abnormalities. It should be clear to radiographers or researchers who are performing the scans, what is expected of them during scan acquisition.

### Review of scans

Traditionally, there has not been any moral obligation for researchers to ‘hunt’ or actively look for incidental findings when conducting research [26, 34]. Many respondents within our interview study did not support a duty to look for abnormalities. By contrast, however, attendants at our expert meeting agreed that it is currently best practice to arrange some form of routine review of research scans. The NIH, for instance, requires that on its campuses, all research scans are “examined by a qualified and experienced member of the research team” [43]. Also the UK Medical Devices Agency (MDA) recommends that all volunteer MRI examinations are routinely reported by a radiologist [18]. The international Mind Research Network provides routine review for its members by contracted board-certified neuroradiologists [44]. Some studies make use of a two-step approach to routine review of scans, including a first step in which trained researchers (radiologists in training) review scans for abnormalities, and a second step in which an expert radiologist clinically reviews suspect scans [29]. Though for some research centres, especially those that are not hospital-based or hospital-affiliated, it may be logistically and/or financially difficult to arrange routine review of scans, the costs need not be prohibitive [44]. Further, when scans acquired are not of diagnostic quality (e.g. fMRI scans), routine review may not be feasible or valuable. However, in large imaging studies in which diagnostic-quality scans are acquired, some form of routine review should be performed [29]. This recommendation was corroborated in our expert meeting. This implies that in some research settings, researchers may be expected to actively look for abnormalities, which raises questions about a ‘duty to hunt’ incidental findings.

### Consultation on detected abnormalities

When an abnormality is detected, the research scan should be assessed by an expert [19], usually a radiologist. The radiologist is consulted first to verify that an abnormality is indeed present on the scan and to provide a diagnosis. Then, the radiologist will have to determine whether or not the abnormality is of clinical relevance to the research participant, and whether or not it should be reported to the research participant. This judgment should preferably be made not by the radiologist alone, but in consultation with a relevant clinician. When a list of findings (not) to report has previously been established (in collaboration with relevant clinicians), this list can serve as a basis for the radiologist’s judgment of clinical significance. To many ethics committees, confirmation by an expert radiologist is an explicit requirement for any feedback of incidental findings to research participants [45]. This requirement is applicable to all research contexts, and implies that even for small-scale scan studies, research teams should establish contact with a radiologist who can be consulted in case of a presumed abnormality. A pathway for consultation needs to be in place prior to the start of the study.

### Communication of the incidental finding

In some countries, the communication of a clinically relevant incidental finding to a research participant must legally be undertaken by a licensed physician. In these countries, one or more physicians should preferably be part of the research team of large-scale imaging studies, in which incidental findings are expected to occur frequently. In small-scale studies or in studies in which only sequences lacking diagnostic utility are acquired, it may suffice for the research team to make prior arrangements with regional primary care physicians or medical specialists, who can be called upon in case an incidental finding is detected. The feedback of incidental findings will then take place via the primary care physician or general practitioner of the research participant [19, 46]. In other jurisdictions, researchers (who are not physicians) may report their findings directly to research participants and advise them to consult with a primary care physician or a medical specialist. Some of our respondents felt that it would violate the privacy rights of the research participant to ‘go behind their backs’ and inform their primary care physicians without informing them first. This has been suggested in other studies, too [1]. Experts attending our meeting stated that therefore, in their studies, research participants are contacted directly and verbally (over the phone) by a physician who is part of the research team. In some countries, other experts indicated, a written letter would be the customary manner in which first contact is made with the research participant about the incidental finding. Most attendants agreed that although serious findings should preferably be discussed face-to-face with the research participant [36], in their experience, it is possible to discuss incidental findings adequately over the course of one or more phone calls, provided that follow-up clinical care is offered in a timely manner.

### Further clinical management and follow-up of the research participant

Attendants at our expert meeting agreed that researchers should take responsibility for the follow-up of the research participant. They are expected to prevent or minimize harm in research participants, but should also show respect for research participants [46]. Moreover, they should provide the ancillary care of returning clinically relevant incidental findings [34]. It can be argued that a certain degree of care entails that researchers cannot merely inform research participants about a potentially significant finding, they must also support them in *addressing* that finding. As researchers themselves may not be in the position to address the finding, they should help research participants find physicians who can. At minimum, researchers must refer the research participant to a relevant clinician, and must make the research scan and/or the radiologist’s report available. Ideally, the research team will have made prior arrangements with relevant clinicians in an associated hospital or elsewhere in the region, such that, whenever an incidental finding is detected, the research participant can be seen in a matter of days. Especially when the finding is serious and/or requires urgent medical attention, clinical follow-up should be available to research participants in a timely manner. The research team should preferably arrange some form of evaluation of the clinical follow-up of research participants, with the aim of further improving the pathway for the handling of incidental findings.

The seven steps described above will interact with one another. For example, the type of scans acquired (i.e. diagnostic-quality or not) will influence the likelihood that clinically relevant incidental findings are detected and therewith, will determine whether or not it is sensible to arrange routine clinical review of research scans. In studies in which diagnostic-quality scans are made, much more demanding pathways for the handling of incidental findings will be required than in studies in which sequences are acquired of limited diagnostic utility. After all, in the former, incidental findings will be frequent and interpretable, and, when clinically relevant, will require action on the part of the researcher. The line of action to be undertaken by researchers who acquire diagnostic-quality research scans, should be carefully anticipated, agreed upon, and assessed by an ethics review committee. Researchers and ethics review committees should increase their focus on the careful handling of incidental findings detected through imaging in research, so that ad hoc decision-making can be avoided and that research participants can know, in advance, what to expect with regard to incidental findings.

## Conclusion

This paper presents an ethical framework consisting of seven steps, in which researchers should make decisions about the design of a pathway for the handling of incidental findings prior to the start of their imaging study. Given the variability among research settings, the framework does not prescribe how - or what - decisions should be made. The framework *does* point out *that* - and where - decisions must be made, and requires researchers to justify these decisions based not only on cost and feasibility considerations, but also on current best practices and research participants’ moral expectations.

Ethics review committees have the important task of evaluating pathways for the detection, management and communication of incidental findings within imaging studies. Whereas, as of yet, ethics committees have commonly required *that* a pathway be in place for the handling of incidental findings, they have not always specified what that pathway should look like. With this framework and the considerations offered in this paper, we aim at assisting ethics committees in the assessment of the appropriateness of proposed pathways. The development of consistent, transparent and appropriate pathways will be indispensable to maintain public trust in the coming years, as research imaging will affect thousands of research participants worldwide. In research contexts in which diagnostic-quality scans are acquired, appropriate pathways for the handling of incidental finding will require additional effort, funding and care on the part of the researchers. This additional effort, however, is not a reason to neglect incidental findings. Rather, it is necessary to meet the principle of reciprocity, as it is part of researchers’ responsibilities to protect the interests of research participants.
